# Supplementary material for: Incidence of Burnout Syndrome among Anesthesiologists and Intensivists in France: The REPAR Study
Source: Int J Environ Res Public Health. 2023 Jan 18;20(3):1771. doi: 10.3390/ijerph20031771 (PMC9914122; doi:10.3390/ijerph20031771)
Supplement: Supplementary file 1 [file ijerph-20-01771-s001.zip › ijerph-2055406-supplementary.pdf]

SUPPLEMENTARY MATERIAL for

ORIGINAL ARTICLE:

Incidence of Burnout Syndrome among Anesthesiologists and Intensivists in France: The REPAR Study

Authors: Barnabé Berger, Pierre-Julien Cungi, Ségolène Arzalier, Thomas Lieutaud, Lionel Velly, Pierre Simeone and Nicolas Bruder

In *International Journal of Environmental Research and Public Health*

TABLE OF CONTENTS

SUPPLEMENTARY FIGURES AND TABLES *3*

Supplementary Figure S1: Distribution of professional profiles. **3**

Supplementary Figure S2: Anesthesiologists and intensivists age distribution and comparison to national data (CNOM 2015). **4**

Supplementary Figure S3: How anesthesiologists and intensivists describe their relations at work. **5**

Supplementary Table S1: Evaluation of anesthesiologists and intensivists work conditions using a self-assessment numerical rating scale from 0 to 10. **6**

Supplementary Table S2: Comparison of different items according to the gender. **7**

Supplementary Table S3: Comparison of different items according to pratice: public versus private. **8**

SUPPLEMENTARY *9*

Supplementary S1. Bos Epidemiology And Risk Factors Identified In The Literature **9**

Supplementary S2. BOS Epidemiology and prevention axis identified in the literature **10**

Supplementary References *11*

SUPPLEMENTARY FIGURES AND TABLES

Supplementary Figure S1: Distribution of professional profiles.

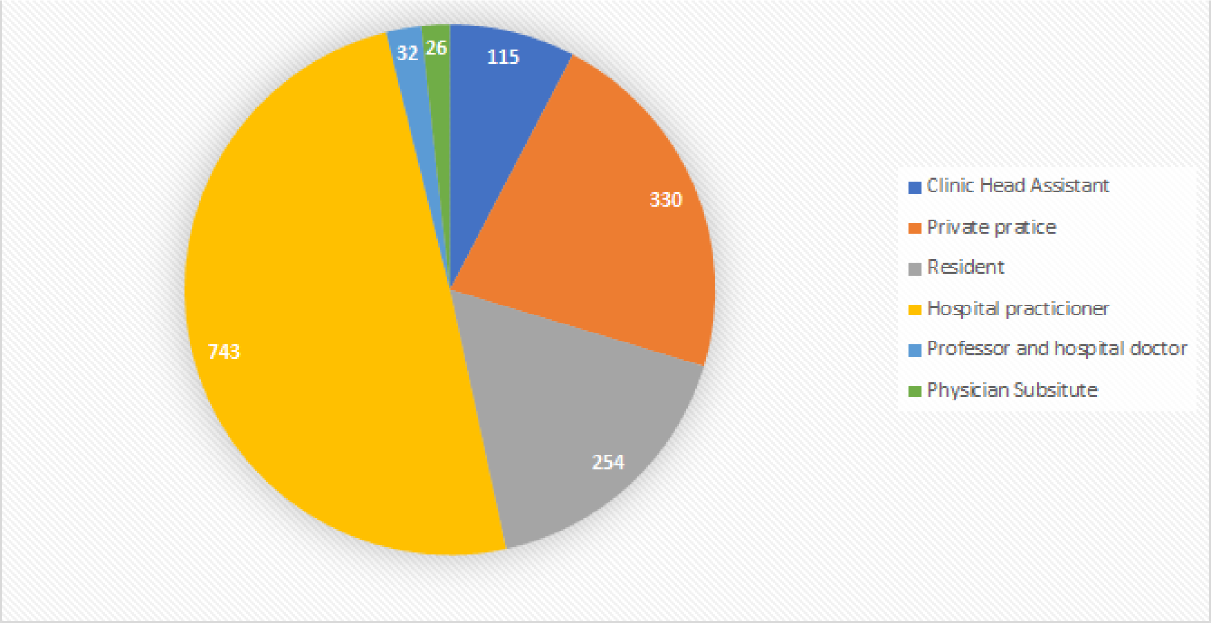

Supplementary Figure S2: Anesthesiologists and intensivists age distribution and comparison to national data (CNOM 2015).

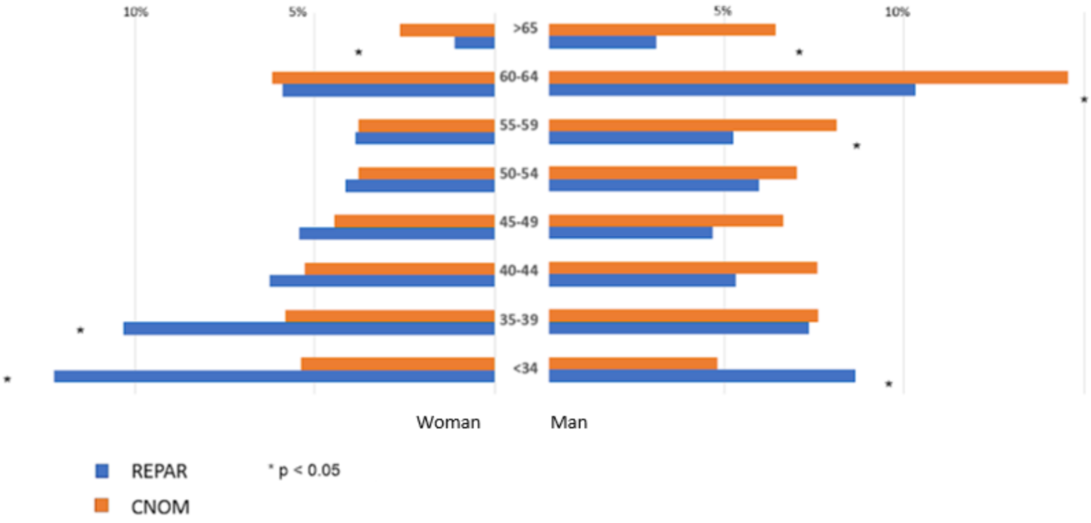

Supplementary Figure S3: How anesthesiologists and intensivists describe their relations at work.

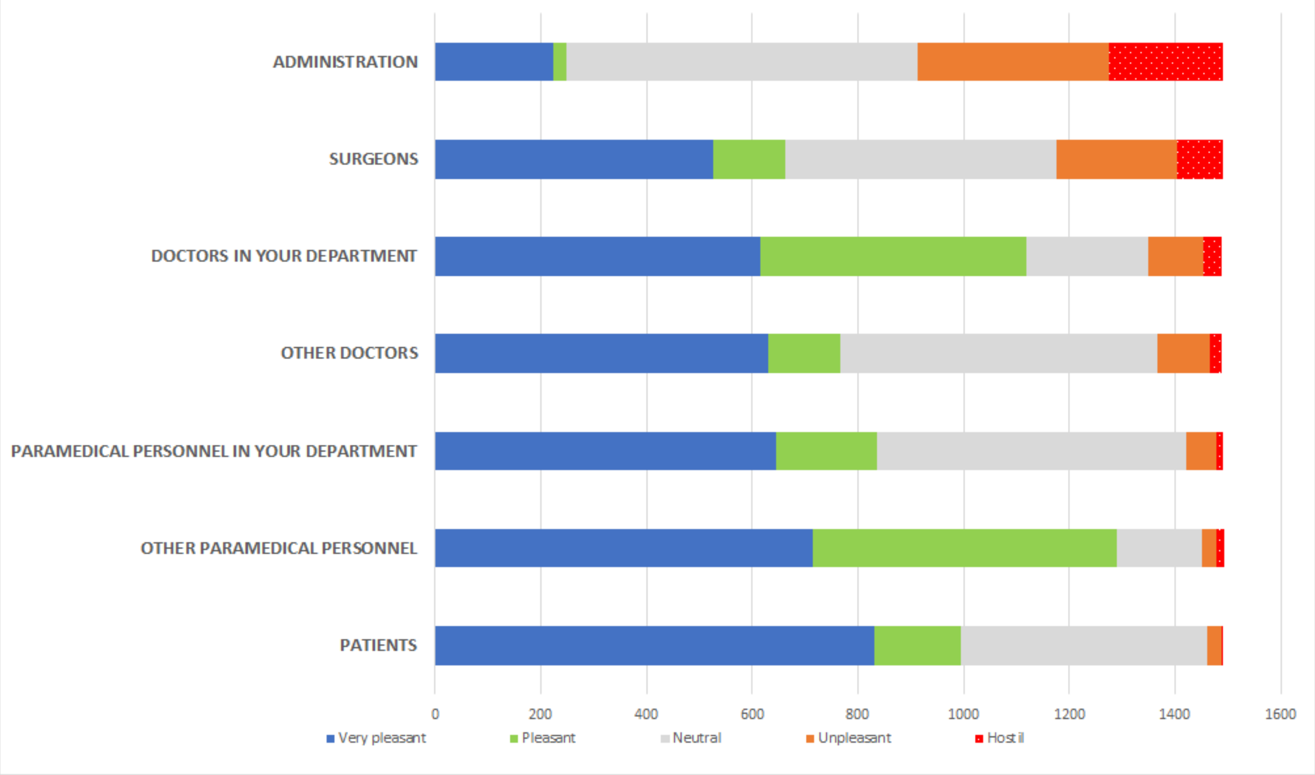

Supplementary Table S1: Evaluation of anesthesiologists and intensivists work conditions using a self-assessment numerical rating scale from 0 to 10.

	Values	Scale > 5, n(%)
Freedom of action at work	7 [5-8]	1000 (67%)
Salary	6 [3-8]	794 (53%)
Material conditions	5 [3-8]	740 (49%)
Way to work	8 [6-10]	1149 (77%)
Personal space at work	6 [3-8]	775 (52%)
Communication at work	8 [6-9]	1130 (75%)
Skills properly recognized	7 [4-8]	957 (64%)

Supplementary Table S2: Comparison of different items according to the gender (*p*<0.05 *) (n=1496).**

	1496 AI known gender		
	Male, (n=775)	Female, (n=721)	*p* < 0.05
**Social characteristics**			
Mean age	45	40	***
Female gender	0%	100%	***
Being in a relationship	87.1%	71.7%	***
Divorced	17.2%	11.4%	***
Medical or paramedical spouse	73.0%	55.6%	***
**CBI score**			
Personal BO	39.03	48.14	***
Work-related BO	37.08	43.54	***
Patient-related BO	22.81	23.74	
Total	32.97	38.47	***
Main criterion	19.1%	30.2%	***
**Life outside work**			
Consulting a psychologist or a psychiatrist on a regular basis	27.3%	40.9%	***
Using psychotropic drugs	6.5%	6.9%	
MDI > 25	7.0%	11.4%	***
MDI mean score	11 ±8	14 ±9	***
Tobacco(> 16 days per month)	12.3%	10.5%	
Alcohol (> 16 days per month)	26.2	17.3	***
Lack of sleep during work periods	61.5%	76.6%	***
Hours of sleep per night	6.6 ±0.9	6.6 ±0.9	
Spare time scale > 5	32.6%	28.0%	
Spare time (without sport)	5.9 ±6.7	4.4 ±6.1	***
Hours dedicated to sport	2.6 ±2.5	1.7 ±1.6	***
Financial difficulties (often/always)	7.5%	6.5%	
Challenge with weight control	36.3%	44.7%	***
BMI	25.0 ±3.5	22.7 ±3.7	***
**Work life**			
Day after night-shift answer	12.90%	12.10%	
Resident (vs senior doctors)	15.30%	18.70%	
Public practice	54.1%	64.8%	***
Mean time working per week	57.7 ±11.9	55.9 ±11.0	***
Mean number of nightshift per month	4.4 ±2.5	4.1 ±1.9	***
Rest period after night shift	68.7%	81.8%	***
Semester off during during residency	11.60%	15.60%	***
Main practice (seniors) : anesthesia / intensive care / both	62.4 / 11.7 / 25.9 %	65.0 / 11.8 / 23.2 %	
Bad relationships with :			
Paramedical personnel in your department	3.0%	3.6%	
Paramedical personnel in other departments	5.4%	5.1%	
Doctors in your department	8.3%	10.4%	
Doctors in other departments	8.5%	9.2%	
Surgeons	22.3%	20.9%	
Patients	2.7%	2.4%	
Administration	39.0%	39.5%	
Many conflicts at work (> 1 per months)	31.9%	35.5%	
Skills properly recognized scale > 5	65.3%	62.8%	
Salary scale > 5	56.9%	48.5%	***
Working material conditions scale > 5	53.0%	45.8%	***
Personal space at work scale > 5	51.1%	56.2%	
Communication equipment at work scale > 5	60.7%	48.2%	
Workteam feeling scale > 5	75.00%	72.20%	
Freedom of action at work scale > 5	73.5%	66.6%	***
Work global situation scale > 5	77.4%	73.2%	
Team size > 30	32.5%	33.4%	
Seniority within the team (years) (median [interquartile])	7 [2-16]	4 [2-10]	
Time interval since last vacations (weeks) (median [interquartile)	6 [3-11]	6 [4-10]	
Lunch break (night shift excluded): often or always	50.1%	43.8%	***
Team meeting (> 1 per month)	49.8%	53.1%	
Time spent on training	50.1%	43.8%	***
Considering changing job or stopping study (now or in the past)	50.3%	58.5%	***

Supplementary Table S3: Comparison of different items according to pratice: public versus private (*p* < 0.05 *) (seniors only, n= 1254).**

	1254 senior AI		
	**Private, (n=356)**	**Public, (n=898)**	***p* < 0.05**
**Social characteristics**			
Mean age	50	44	***
Female gender	33.4%	52.0%	***
Being in a relationship	86.8%	81.2%	***
Divorced	25.1%	12.4%	***
Medical or paramedical spouse	88.8%	69.7%	***
**CBI score**			
Personal BO	39.6	44.2	***
Work-related BO	37.2	41.2	***
Patient-related BO	24.1	23.2	
Total	33.6	36.2	***
Main criterion	20%	26%	***
**Life outside work**			
Consulting a psychologist or a psychiatrist on a regular basis	35.5%	35.7%	
Using psychotropic drugs	10.1%	6.7%	***
MDI > 25	5.1%	8.7%	***
MDI mean score	10 ±8	13 ±9	***
Tobacco(> 16 days per month)	7.60%	11.10%	
Alcohol (> 16 days per month)	23.90%	19.60%	
Lack of sleep during work periods	64.5%	68.2%	
Hours of sleep per night	6.5 ±0.9	6.6 ±0.9	
Spare time scale > 5	44.4%	26.7%	***
Spare time (without sport)	5.7 ±6.7	4.9 ±6.4	
Hours dedicated to sport	2.4 ±2.3	2.1 ±2.2	***
Financial difficulties (often/always)	8.1%	6.2%	
Challenge with weight control	34.8%	42.9%	***
BMI	24.4 ±3.2	24.1 ± 4.1	
**Work life**			
Day after night-shift answer	8.4%	12.7%	***
Resident (vs senior doctors)	0	0	
Public practice	0%	100%	***
Mean time working per week	54.4 ±12.9	56.3 ±10.8	***
Mean number of nightshift per month	3.8 ±2.8	4.3 ±2.1	***
Rest period after night shift	40.4%	83.1%	***
Semester off during during residency	6.90%	9.70%	
Main practice (seniors) : anesthesia / intensive care / both	83.0 / 1.4 / 15.7%	63.8 / 15.6 / 20.6%	***
Bad relationships with :			
Paramedical personnel in your department	0.8%	3.1%	***
Paramedical personnel in other departments	2.5%	4.7%	
Doctors in your department	7.3%	10.7%	
Doctors in other departments	8.1%	8.1%	
Surgeons	20.8%	22.0%	
Patients	3.1%	1.9%	
Administration	38.8%	38.0%	
Many conflicts at work (> 1 per months)	27.2%	37.5%	***
Skills properly recognized scale > 5	71.9%	61.9%	***
Salary scale > 5	82.9%	45.5%	***
Working material conditions scale > 5	60.7%	48.2%	***
Personal space at work scale > 5	51.1%	56.2%	
Communication equipment at work scale > 5	82.5%	76.5%	***
Workteam feeling scale > 5	76.7%	71.4%	
Freedom of action at work scale > 5	81.3%	72.0%	***
Work global situation scale > 5	74.4%	61.9%	***
Team size > 30	35.8%	32.6%	
Seniority within the team (years) (median [interquartile]	10 [4-20]	6 [3 -14]	
Time interval since last vacations (weeks) (median [interquartile])	5 [3-8]	6 [4-11]	***
Lunch break (night shift excluded): often or always	33.10%	48.50%	***
Team meeting (> 1 per month)	32.3%	57.8%	***
Time spent on training	33.1%	48.5%	***
Considering changing job or stopping study (now or in the past)	50.0%	56.4%	***

SUPPLEMENTARY

Supplementary S1. BOS Epidemiology and risk factors identified in the literature.

Identified	Female caregivers [1–3]
	Residents / young practitioners [1,2,4]
	Conflicts : surgeons, patients, colleagues, family [2,5]
	Excessive workload [2,6,7]
Suspected	Lack of recognition [3]
	Lack of independence [3,8]
	Unsatisfactory salary [2]
	Big size team [1]
	Emotional profile and personality [9]
	Genetic factors [10]
	Cultural factors : physician wellness taboo [6,11]
	Unhealthy way of life [6]
	Lack of sleep [12,13]
	Lack of physical activity [14]

Factors were said "identified" when they were studied in interventional studies, meta analysis, or observational studies only including french AI on a national scale.

Supplementary S2. BOS Epidemiology and prevention axis identified in the literature.

ORGANIZATIONAL	Identified	Duty-hour restriction (< 80 h / week) [15,16]<br>Better organization in working time [17]<br>Team meetings [17]
	Suspected	Flexibility in working time [18,19]<br>Optimizing night shift schedule [20]<br>Foster communication among teams [18,19,21] :<br>- Meetings : morbi-mortality, protocols, teaching<br>- Parrainages, activités, cohésion [3]<br>- Premises [21], phones, softwares [22]<br>- Small teams [1].<br>Occupational health-care development [23,24]<br>Evaluation program [20] : former decisions evaluation, new technologies, changes in work environment<br>AI training [19] : dedicated time, evaluate institutional need and practitioner wish<br>Reducing paper work [21,22]
INDIVIDUAL	Identified	Discussion groups [15]<br>Educational interventions targeting physicians' self confidence or communication skills [17]
	Suspected	Lifestyle modification: « *self-care* », sport, sleep, nutrition, spare time [6,14]<br>Information about BO, how to identify and how to manage it [23]<br>Specialized care of depression, cessation of work if needed [23]<br>Stress management training : mindfullness, self-compassion, resilience, empathy [17,18,25,26]

Factors were said "identified" when they were studied in interventional studies, meta analysis, or observational studies only including french AI on a national scale.

Supplementary References

[1] Lapa TA, Madeira FM, Viana JS P-GJ. Burnout syndrome and wellbeing in anesthesiologists: the importance of emotion regulation strategies. Minerva Anestesiol 2017.
[2] Estryn-behar M. Comment des médecins hospitaliers apprécient leurs conditions de travail . Réponses aux questions ouvertes d ' une enquête nationale. Revue Française Des Affaires Sociales 2010;4:27–52.
[3] Dureuil B. Comment l ' organisation du travail peut prévenir l ' épuisement professionnel en anesthésie-réanimation ? 2011.
[4] Panagioti M, Panagopoulou E, Bower P, Lewith G, Kontopantelis E, Chew-Graham C, et al. Controlled Interventions to Reduce Burnout in Physicians. JAMA Internal Medicine 2017. https://doi.org/10.1001/jamainternmed.2016.7674.
[5] Heslin MJ, Doster BE, Daily SL, Waldrum MR, Boudreaux AM, Smith AB, et al. Durable Improvements in Efficiency, Safety, and Satisfaction in the Operating Room. Journal of the American College of Surgeons 2008;206:1083–9. https://doi.org/10.1016/j.jamcollsurg.2008.02.006.
[6] Williams D, Tricomi G, Gupta J, Janise A. Efficacy of burnout interventions in the medical education pipeline. Academic Psychiatry 2015. https://doi.org/10.1007/s40596-014-0197-5.
[7] Busireddy KR, Miller JA, Ellison K et al. Efficacy of Interventions to Reduce Resident Physician Burnout: A Systematic Review. Grad Med Educ 2017;9:294–301.
[8] Shapard EWBL. Employee Burnout: A Meta-Analysis of the Relationship Between Age or Years of Experience. Human Resource Development Review 2004 2004;3(2):102-2.
[9] Doppia MA, Estryn-Béhar M, Fry C, Guetarni K, Lieutaud T. Enquête comparative sur le syndrome d'épuisement professionnel chez les anesthésistes réanimateurs et les autres praticiens des hôpitaux publics en France (enquête SESMAT). Annales françaises d'Anesthesie et de Réanimation 2011;30:782–94. https://doi.org/10.1016/j.annfar.2011.05.011.
[10] Mion G, Libert N, Journois D. Facteurs associés au burnout en anesthésie-réanimation. Enquête 2009 de la Société française d'anesthésie et de réanimation. Annales françaises d'Anesthesie et de Réanimation 2013;32:175–88. https://doi.org/10.1016/j.annfar.2012.12.004.
[11] Howard SK, Rosekind MR, Katz JD, Berry AJ. Fatigue in Anesthesia Implications and Strategies for Patient and Provider Safety. Anesthesiology 2002;97:1281–94. https://doi.org/10.1097/00000542-200211000-00035.
[12] Bodenheimer T, Sinsky C. From triple to Quadruple Aim: Care of the patient requires care of the provider. Annals of Family Medicine 2014. https://doi.org/10.1370/afm.1713.
[13] Blom V, Bergström G, Hallsten L, Bodin L, Svedberg P. Genetic susceptibility to burnout in a Swedish twin cohort. European Journal of Epidemiology 2012;27:225–31. https://doi.org/10.1007/s10654-012-9661-2.
[14] Embriaco N, Azoulay E, Barrau K, Kentish N, Pochard F, Loundou A, et al. High level of burnout in intensivists: Prevalence and associated factors. American Journal of Respiratory and Critical Care Medicine 2007;175:686–92. https://doi.org/10.1164/rccm.200608-1184OC.

[15] Zwack J, Schweitzer J. If Every Fifth Physician Is Affected by Burnout, What About the Other Four? Resilience Strategies of Experienced Physicians. Academic Medicine 2013. https://doi.org/10.1097/ACM.0b013e318281696b.
[16] West CP, Dyrbye LN, Erwin PJ, Shanafelt TD. Interventions to prevent and reduce physician burnout: a systematic review and meta-analysis. The Lancet 2016;388:2272–81. https://doi.org/10.1016/S0140-6736(16)31279-X.
[17] Chiron B, Michinov E, Olivier-Chiron E, Laffon M, Rusch E. Job Satisfaction, Life Satisfaction and Burnout in French Anaesthetists. Journal of Health Psychology 2010. https://doi.org/10.1177/1359105309360072.
[18] Kluger MT, Townend K, Laidlaw T. Job satisfaction, stress and burnout in Australian specialist anaesthetists. Anaesthesia 2003;58:339–45. https://doi.org/10.1046/j.1365-2044.2003.03085.x.
[19] Leriche B. Le Médecin Malade. Conseil national de l'ordre des médecins; 2008.
[20] Wallace JE, Lemaire JB, Ghali WA. Physician wellness: a missing quality indicator. The Lancet 2009. https://doi.org/10.1016/S0140-6736(09)61424-0.
[21] CFAR. Providing online self-tests to identify or assess behavior or health disorders related to chronic stress at work by health professionals in anesthesiology and intensive care in France. 2015.
[22] HAS. Repérage et prise en charge cliniques du syndrome d'épuisement professionnel ou burnout 2017:148.
[23] Morais A, Maia P, Azevedo A, Amaral C, Tavares J. Stress and burnout among Portuguese anaesthesiologists. European Journal of Anaesthesiology 2006;23:433–9. https://doi.org/10.1017/S0265021505001882.
[24] McManus I, Keeling A, Paice E. Stress, burnout and doctors' attitudes to work are determined by personality and learning style: A twelve year longitudinal study of UK medical graduates. BMC Medicine 2004;2:29. https://doi.org/10.1186/1741-7015-2-29.
[25] Bria M, Baban A, Dumitrascu D. Systematic review of burnout risk factors among European healthcare professionals. Cognition, Brain, Behavior: An Interdisciplinary Journal 2013;16:423–52.
[26] Lindwall M, Gerber M, Jonsdottir IH, Börjesson M, Ahlborg G. The relationships of change in physical activity with change in depression, anxiety, and burnout: A longitudinal study of Swedish healthcare workers. Health Psychology 2014. https://doi.org/10.1037/a0034402.
